# Antigen-aware TCR embeddings reveal conserved immune patterns for personalized immune monitoring

**DOI:** 10.64898/2026.10.09.757444

**Published:** 2026-10-16

**Authors:** Mingyao Pan, Jing Hu, Yizhou Tracy Wang, Yuhao Tan, Hailong Hu, Ziqi Yang, Julia Fleming, Yuanyuan Pang, Bo Li

## Abstract

T cell receptor (TCR) repertoires are highly individualized, while technical variation further complicates comparisons across individuals and cohorts. Here, we present TCRmeta, an antigen-aware framework that integrates population-scale repertoire relationships with experimentally defined antigen specificity to establish a shared space for repertoire analysis. TCRmeta predicted mutation-induced changes and captured antigen-related relationships in TCR specificity. Despite extensive sequence diversity, TCRmeta revealed a conserved repertoire landscape, enabling construction of a healthy reference for quantifying repertoire dispersal. Across cohorts, aging was associated with conserved V gene changes and progressive dispersal, with greater dispersal in CMV-positive individuals. Longitudinal analysis showed that repertoires progressively shifted toward the healthy reference following bone marrow transplantation or antiretroviral therapy. To directly compare repertoires in TCRmeta space, we developed Energy Shift, a pairwise measure robust to technical variation and HLA background while remaining sensitive to biological differences. Together, TCRmeta enables quantitative cross-individual and longitudinal immune monitoring.

**Teaser:**

TCRmeta reveals conserved organization underlying highly individualized TCR repertoires and enables robust tracking of repertoire changes across immune states.

